# Biocoder: A programming language for standardizing and automating biology protocols

**DOI:** 10.1186/1754-1611-4-13

**Published:** 2010-11-08

**Authors:** Vaishnavi Ananthanarayanan, William Thies

**Affiliations:** 1 Max Planck Institute for Molecular Cell Biology and Genetics, Pfotenhauerstrasse 108, 01307 Dresden, Germany; 2 Microsoft Research India, 196/36 2nd Main, Sadashivanagar, Bangalore, 560080, India

## Abstract

**Background:**

Published descriptions of biology protocols are often ambiguous and incomplete, making them difficult to replicate in other laboratories. However, there is increasing benefit to formalizing the descriptions of protocols, as laboratory automation systems (such as microfluidic chips) are becoming increasingly capable of executing them. Our goal in this paper is to improve both the reproducibility and automation of biology experiments by using a programming language to express the precise series of steps taken.

**Results:**

We have developed BioCoder, a C++ library that enables biologists to express the exact steps needed to execute a protocol. In addition to being suitable for automation, BioCoder converts the code into a readable, English-language description for use by biologists. We have implemented over 65 protocols in BioCoder; the most complex of these was successfully executed by a biologist in the laboratory using BioCoder as the only reference. We argue that BioCoder exposes and resolves ambiguities in existing protocols, and could provide the software foundations for future automation platforms. BioCoder is freely available for download at http://research.microsoft.com/en-us/um/india/projects/biocoder/.

**Conclusions:**

BioCoder represents the first practical programming system for standardizing and automating biology protocols. Our vision is to change the way that experimental methods are communicated: rather than publishing a written account of the protocols used, researchers will simply publish the code. Our experience suggests that this practice is tractable and offers many benefits. We invite other researchers to leverage BioCoder to improve the precision and completeness of their protocols, and also to adapt and extend BioCoder to new domains.

## Background

For decades, biologists have relied on written descriptions of protocols to guide their experiments in the laboratory. However, due to recent technology trends, the practice of describing protocols with free-flowing English-language text is quickly becoming inadequate and obsolete. First, we are witnessing immense advances in laboratory automation systems. The increasing density of microfluidic devices has been compared to Moore's Law [1,2], with recent products supporting up to 9,216 parallel reactions [3]. In order to leverage such technologies for biological experimentation, it will be necessary to express the protocols in a format that is not only comprehensible by humans, but also by machines. Second, the complexity of biology protocols is increasing dramatically. As fields such as synthetic biology attempt to synthesize living systems as a composition of many parts, we will need to execute lengthy protocols with great precision. It will become imperative for researchers to share complete descriptions of their methods in a form that can be consistently replicated in other laboratories.

Unfortunately, today's descriptions of biology protocols are rarely suitable for either reproducibility or automation. One of the most glaring problems is that of incompleteness: methods are described in the literature without providing a complete and self-contained account of the steps taken. For example, a recent protocol [4] indices that methods for an electrophoretic mobility shift assay (EMSA) were "as described previously". While a citation is given, the referenced paper [5] cites another [6], which cites another [7], which cites another [8], each time saying that the method in question was "as described previously". Only after following four references, and going back almost 15 years, is the alleged protocol uncovered - and often there are undocumented modifications along the way.

We are not the first to call for standardization of protocol descriptions. In recent work, Soldatova et al. [9] make an excellent case for the need to formalize biology protocols and propose an ontology called EXACT for doing so. EXACT offers a detailed breakdown of each action in the laboratory, resulting in a description that is considerably more verbose than the original (we offer a detailed comparison to EXACT later). The MIBBI project (Minimal Information for Biological and Biomedical Investigations) develops a set of checklists that represent the minimal information needed to capture various classes of experiments [10]. The journal *BMC Bioinformatics *recommends that authors follow the MIBBI guidelines in reporting their protocols. While specific standards such as MIAME (Minimal Information About a Microarray Experiment) have gained considerable traction, there does not exist a widely used MI-standard for the less structured domain of molecular biology protocols, which is our focus in this paper. There are also several standardization efforts underway in the context of systems biology [11] and synthetic biology [12], though the current focus of these communities is in standardizing the descriptions of genes, models, and biological parts rather than experimental protocols.

There has also been prior work on formalizing protocols for the sake of automation. King et al. [13] developed a "robot scientist" that directs laboratory experiments using a high-level programming language; however, the language does not aim to be portable to automation platforms other than the one used in the research. In prior work, we developed a simple programming language for toy protocols based on mixing and storage, and mapped it to diverse microfluidic devices [14]; however, it is not powerful enough to describe realistic protocols. Amin et al. [15] built on this work in proposing AquaCore, an architecture for microfluidics that includes an instruction set architecture (ISA) for expressing real protocols. However, like the robot scientist, it is specialized for execution on a single platform, and does not express the logical steps needed to reproduce an experiment in another laboratory.

In this paper, we advance this body of work by proposing (to our knowledge) the first programming language that can describe realistic biology protocols while focusing only on their logical functionality, rather than their mapping to any given machine. In other words, the language serves the same role as ontologies such as EXACT and MIBBI in standardizing the description of protocols, though it is also suitable for automation. As a platform for standardizing protocols, a programming language has many advantages over a checklist or an ontology. It allows the use of standard modularity mechanisms, such as procedures, classes, and packages, to define reusable steps that can be called from multiple protocols (we demonstrate such reuse in our results). One can naturally parameterize a program, enabling different configurations to be activated with very small changes. Programs admit simple checks, such as type checking, which can ensure that protocols are well-constructed. Many scientists are already familiar with the basic principles and practice of programming, and have access to programming tools (for editing, documenting, testing, debugging, etc.) that would otherwise need to be built from scratch. Finally, programs provide the shortest path to automation, as they can potentially run on emerging automation platforms. They are also straightforward to simulate for testing and educational purposes on an ordinary computer.

Our vision is to change the way that scientific protocols are communicated: in scholarly publications, can we replace the English-language description of experimental methods with a computer program? Scientists could then download the code and easily replicate the experiment, either via automatic execution or translation to human-readable steps.

## Results and Discussion

**Figure 1:**
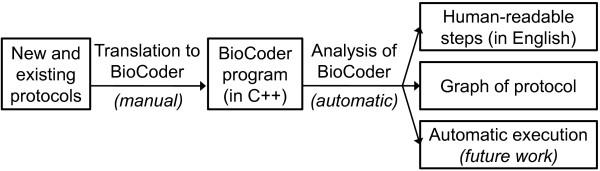
**Overview of the BioCoder system**.

An overview of our system, which is called BioCoder, appears in Figure 1. BioCoder is implemented as a C++ library, enabling users to author new and existing protocols by translating them into a stylized C++ program. In order to make the coded protocols useful in the laboratory, BioCoder provides an automatic translation of the program to a human-readable, English-language sequence of steps (similar to the original protocol description, but with more standardized terminology). It also emits a graph of the protocol to help users visualize the steps. A detailed tutorial on how to use BioCoder is available online [16]. While BioCoder is also suitable for automation, this has yet to be demonstrated, and in this paper we focus on its use in standardization.

We proceed by describing the details of the BioCoder language, as well as our efforts to validate that the language provides an accurate representation of real biology protocols. We have implemented 66 protocols in Biocoder; for the most complex of these protocols (manipulation of plant DNA), we demonstrate an end-to-end, real-world usage scenario whereby a biologist performed the protocol (previously unfamiliar to her) using the BioCoder-generated steps as her only reference. To the best of our knowledge, this represents the first human execution of a protocol that originated from a standardized, programmed description. We also validate the usability of BioCoder as a programming tool via a case study of two undergraduates who were previously unfamiliar with the language. We close by discussing practical applications of BioCoder, from identifying and resolving ambiguous protocols, to its role in automation, laboratory information systems, and education.

### The BioCoder Language

In this section we describe the details of the BioCoder language. We start with an overview of the system, before highlighting the most interesting aspects of the design: standardizing ad-hoc language, separating comments from instructions, supporting symbolic volumes, improving readability of text output, and timing constraints. We close with a brief discussion of the implementatin of BioCoder itself.

#### Overview

The ultimate goal of BioCoder is to enable biologists to express any protocol in a complete and unambiguous way, that is suitable both for automation (by machines) as well as manual execution (by humans). As a first step towards this vision, in this paper we limit our scope in two ways. First, we focus only on the domain of molecular biology, rather than encompassing the full complexity of arbitrary protocols. Second, as it remains an active area of research to integrate all of the functions of a biology lab in an automated machine, we focus our attention on improving the reproducibility of experiments by human biologists. This allows us to decouple our language research from the progress of laboratory automation systems (though we do review early results towards automating the language in a later section).

The design of BioCoder is driven by two rules. First, protocols written in the language should not depend on the setup of any given laboratory. They should describe the logical steps needed, rather than specific locations or pieces of equipment ("fume hood A"). Comparable to "write-once, run anywhere" programming languages such as Java, which are portable between diverse computer platforms, this rule ensures that protocols can be easily reproduced in different laboratories. Second, the language should be suitable for automation (when hardware support is available). This implies that all of the information needed to execute the protocol should be precisely encoded in BioCoder instructions alone. The program should be free of any English-language text that would require human interpretation for correct execution.

One of the first decisions facing any designer of a domain-specific language is whether to build the language from scratch, or whether to embed it within an existing programming language [17]. Both approaches have their merits; while defining a fresh language allows customizing the syntax and semantics to the domain of interest, piggybacking on another language leverages programmers' existing familiarity with that language and allows interoperation with existing libraries and tools. As we anticipate that learning a new language would represent a barrier for many biologists, we choose to embed BioCoder within an existing language: C++. (Users of BioCoder do not need to understand the complex features of C++; our code is legal C with the exception of function overloading, which simplifies programming.) We choose C/C++ because they remain the 1st and 3rd most popular languages in the world today [18], and C is often the first language of instruction in a collegiate environment. While it could also be useful to port BioCoder to other language environments (such as Perl or Python), this exercise would be straightforward given the ideas developed in this paper. Our design decisions and lessons learned are independent from the C/C++ environment.

**Figure 2:**
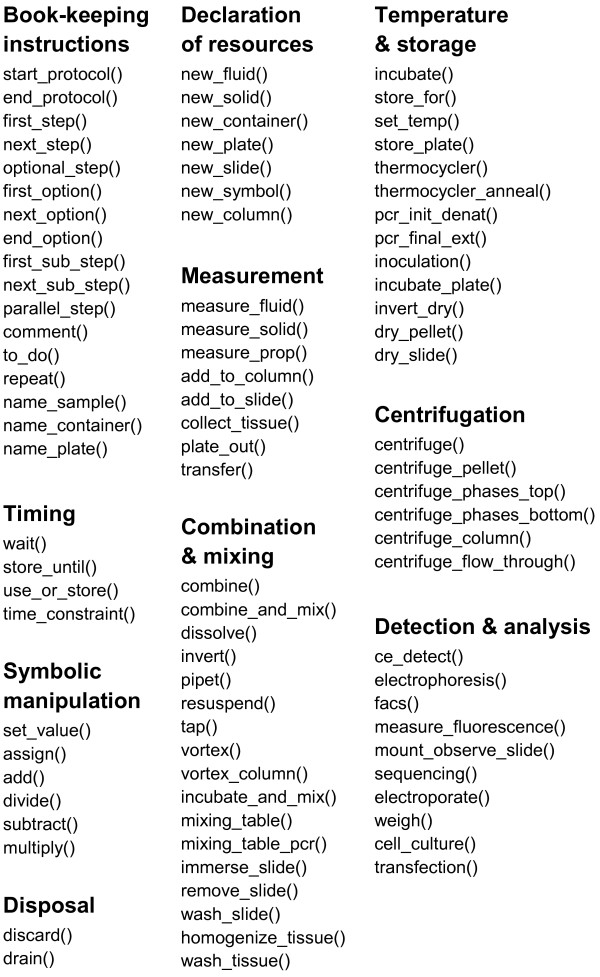
**BioCoder instructions**. Function parameters are not shown.

In embedding BioCoder within C++, we borrow all of the control flow and modularity mechanisms of C++ while assigning special meaning to a set of BioCoder-specific functions (see Figure 2). In this sense, the current version of BioCoder can be thought of as a library rather than a language, though we prefer to think of it as a language as it provides a self-contained vocabulary for describing the steps of a biology experiment. We devote the rest of this paper to the BioCoder functions, which are used to describe the steps of a biology procedure. Users of BioCoder can of course leverage the additional constructs of C++ to achieve goals such parameterization, functional abstraction, etc., which are key to enhancing the scalability, readability, and reusability of the protocols.

##### Example BioCoder program

**Figure 3:**
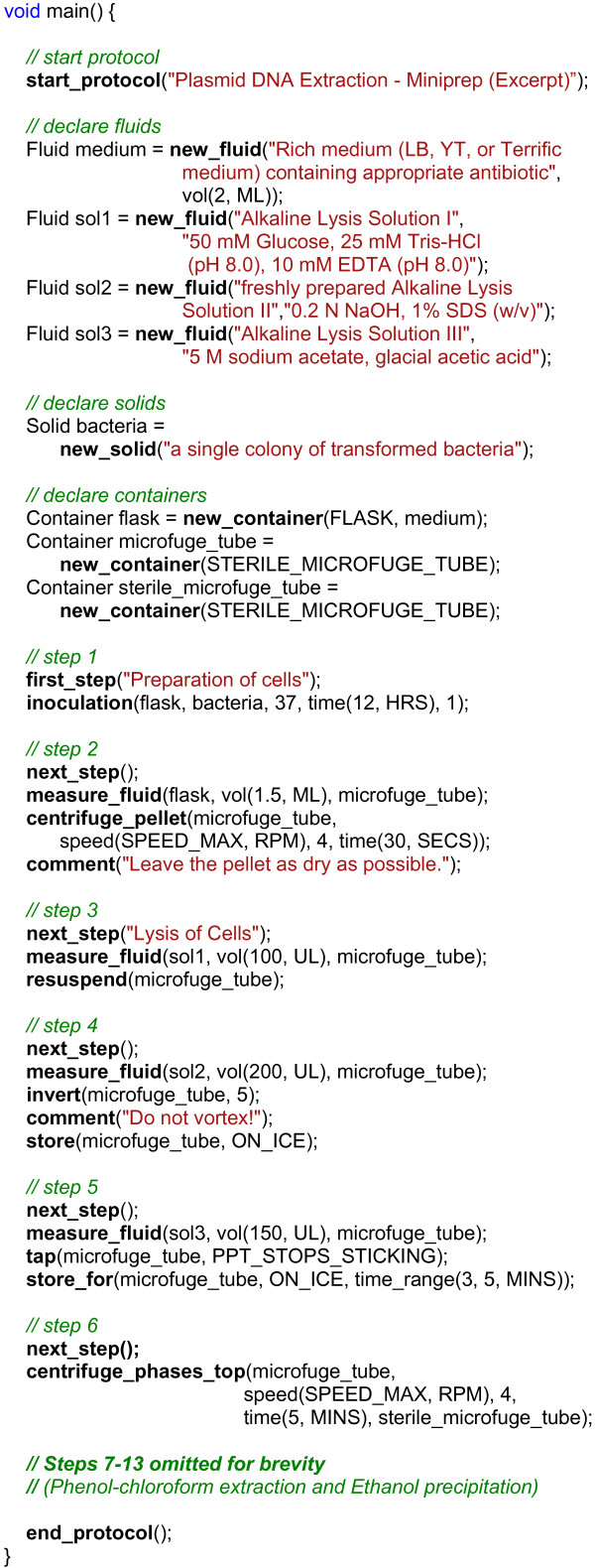
**BioCoder example**. Example BioCoder code for plasmid DNA extraction (miniprep). Steps 7-13 are omitted for brevity.

**Figure 4:**
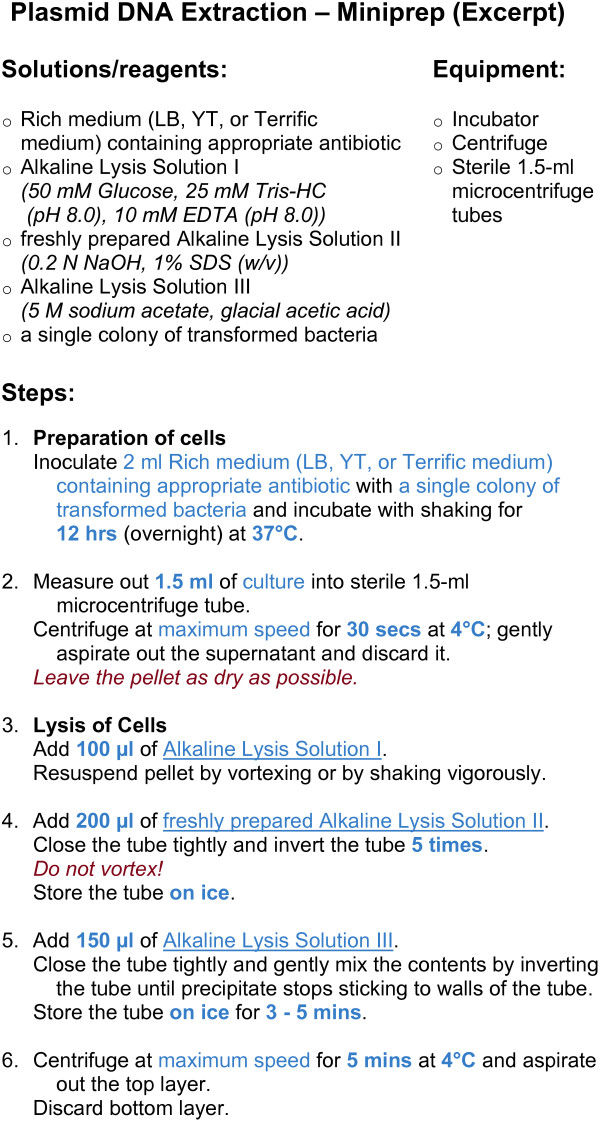
**BioCoder text output**. Auto-generated description of a plasmid DNA extraction protocol, which was produced from the BioCoder code in Figure 3.

**Figure 5:**
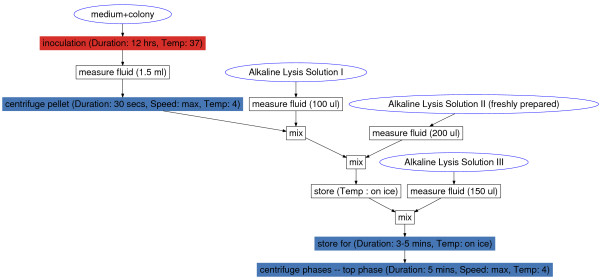
**BioCoder graphical output**. Auto-generated task graph of a plasmid DNA extraction protocol, based on the BioCoder code in Figure 3. Tasks are colored according to the amount of time required to complete them (warmer colors take more time).

The core functions of BioCoder are tabulated in Figure 2. Because detailed documentation for each instruction is available online [19], here we introduce the language by way of an example. Figure 3 shows an excerpt from a protocol for plasmid DNA extraction [20]. The program is written in ordinary C++, and links to a BioCoder library. The BioCoder analysis (implemented as a runtime system) converts the program to a sequence of human-readable instructions (Figure 4) as well as a graphical visualization of the protocol (Figure 5).

The example program (Figure 3) starts by declaring the materials that are used throughout the protocol. These include biological substances such as fluids (reagents, solutions, etc.) and solids (cells, powders, etc.), as well as objects such as containers, plates, slides, and columns. Fluids are most commonly declared without a volume, in which case they represent a renewable stock. A fixed volume of fluid can be isolated by measuring it into a container. Thereafter, all operations on the fluid sample are written in terms of the container; for example, the centrifuge command has an argument of type Container, but not of type Fluid. Manipulating containers as the primary objects of interest allows the system to generate more readable instructions for use in the laboratory. However, in an automated platform, containers may not have a direct analog, in which case they can be ignored by the runtime system and treated merely as wrappers for fluids of interest. Automated platforms such as microfluidic chips may also require different volumes than human operators; this is made possible by using symbolic volumes (detailed later).

The text output (Figure 4) of BioCoder represents an equivalent, human-readable version of the underlying protocol. Only BioCoder instructions are translated to English; if the original program also includes native C++ instructions (such as loops, if statements, arithmetic, etc.), they will be executed during the translation to BioCoder and will not appear in the text version. This allows programmers to use a single, parameterized program to emit multiple variations of a protocol in English.

BioCoder's graphical output (Figure 5) illustrates all of the operations in the protocol as well as the dependences between them. The graph can optionally include intermediate nodes for each container, as well (though this is disabled, for brevity, in the figure). Operations are colored according to the estimated time required to perform them; warmer colors indicate more time-consuming steps.

The BioCoder system represents a work in progress, and we look forward to engaging the broader community in guiding its future direction. Developing a standard for biology protocols will require a concerted effort on the part of many institutions, and we are committed to an open development process. We have released the source code for BioCoder online [19].

In the remainder of this section, we highlight some of the most interesting aspects of BioCoder, with an eye towards generalizable techniques that may also apply to future language designs.

#### Standardizing Ad-Hoc Language

One of the challenges of formalizing biology protocols is translating casual, ad-hoc language into a precise and quantitative scale, while maintaining the flexibility inherent in the original protocol. One example is mixing: there are many words for "mix" in the English language, and few of them have escaped mention in the pages of a biology journal. To address this, we establish a numeric scale for mixing intensity, with each point corresponding to one or more common actions: 1) tap, 2) stir, 3) invert, and 4) vortex/resuspend/dissolve. In other words, we judge the differences between the first three actions (tap, stir, invert) to be significant, while the other actions (vortex, resuspend, dissolve) are interchangeable with respect to mixing intensity. This four-point scale is used in every call to the combine_and_mix instruction in BioCoder. (For convenience, BioCoder also provides separate instructions for tapping, vortexing, and so on, but they are also translated to this scale.) If a vendor is implementing runtime support for BioCoder on an automated platform, they must respect the contract that level-1 mixing (for example) is comparable in intensity to tapping a tube.

A similar approach is needed for phrases involving temperature ("ice cold", "on ice", "boiling water", etc.) as well as time (e.g., "overnight"). A key aspect of translating ambiguous language to a quantitative scale is that it is important not to overly constrain the specification of the program. If the original protocol could tolerate small variations in temperature or time, then the person or machine that is executing the protocol should enjoy the same flexibility.

#### Separating Comments from Instructions

One interesting difference between the description of biology protocols and the description of computer programs is that, in the current descriptions of protocols, many statements are non-essential to the successful completion of the experiment. Rather, the core instructions are interleaved with explanations, warnings, and reminders that are useful for humans, but could be safely ignored if one was following the real instructions as precisely as a computer.

For example, consider the phrase "do not vortex!", which appears frequently in biology protocols, including step 4 of our running example. How does one formalize this instruction in a program? While we could simply discard the statement for the sake of automation, we also aim to foster adoption for realistic use by biologists, who might prefer not to drop such language from the text instructions emitted by BioCoder (especially if it aids in reproducing an experiment).

Our approach to this issue is to separate the concepts of *instructions *(which are needed even for a flawless machine to successfully complete the protocol) and *comments *(which are non-essential). Comments are indicated using the BioCoder comment function, which takes a free-form English language string. This does not violate our original design rules, as this string can be ignored from the standpoint of automation.

Still, one of the principal challenges in translating protocols to BioCoder is drawing the line between instructions and comments. For example, in step 2 of the miniprep protocol (Figure 3), the textbook source prescribes to "leave the pellet as dry as possible". While this statement sounds helpful, since the preceding centrifugation and aspiration should have the same effect, we consider it to be non-essential and thus implement it as a comment.

#### Symbolic Volumes

It is common for biology protocols to contain symbolic volumes, where the experimenter is expected to calculate the actual volumes needed based on the specific circumstances at the time. While such calculations can easily be embedded within a computer program, extra care is needed to preserve the symbolic elements when translating back to an English-readable description.

**Figure 6:**
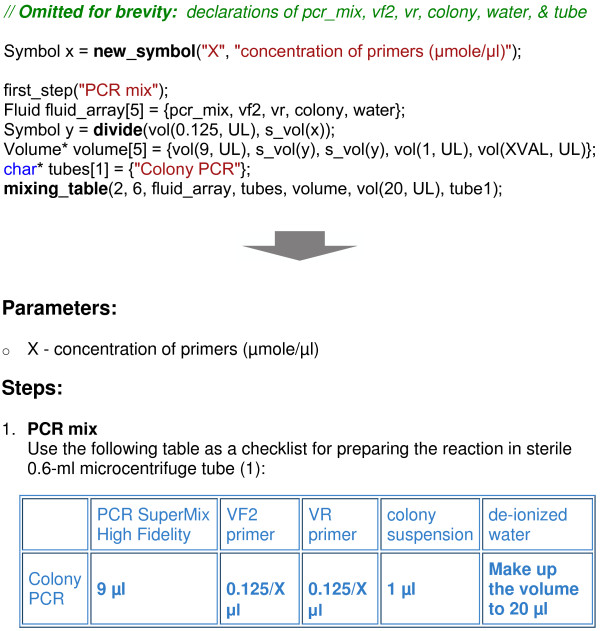
**BioCoder example**. Example of symbolic volumes and high-level mixing instructions in BioCoder. The BioCoder code is shown at top, and the auto-generated text version is shown at bottom.

To address this issue, BioCoder includes a type Symbol that represents an unknown quantity. Variables of this type can optionally be assigned a numeric value, for the sake of automatic execution and also for generating text instructions for a specific values of the parameters. However, if no value is assigned, the variable appears as a symbol throughout the emitted instructions. BioCoder also supports arithmetic on symbols (via predefined functions such as add and subtract), which lead to symbolic formulas in the English-language description. An example of this functionality appears in Figure 6.

Symbolic volumes could also allow a single program to seamlessly transfer between automation platforms that have different capacities for fluids (e.g., a macro- and micro-fluidic device). If all symbolic volumes are defined in proportion to a baseline symbol, then the machine could scale the value of that free variable such that all of the fluid volumes in the program are a good match for the machine. Such an analysis could rely on automatic volume management [21] to estimate the overall volume demands of a program.

#### Improving the Readability of Text Output

Much of the effort that we invested in BioCoder went towards ensuring that the English-language output closely resembles that of a standard biology protocol. The translation works as a runtime system, in which the program is executed and each call to a BioCoder function generates a corresponding text output. This implies that ordinary C++ operations (such as control flow and arithmetic) are evaluated once, during the translation to text, rather than being emitted as textual instructions for the biologist to follow. For example, if one writes a loop (from 1 to 10) around a BioCoder instruction, then that instruction will be emitted ten times in the textual version, rather than preserving the loop construct in the text. While we considered extending BioCoder with control flow instructions (such as loops and if/then/else statements), we did not find any application of such instructions across our benchmark suite. Instead, the common case is that a protocol is parameterized according to a given condition (e.g., gram-positive or gram-negative bacteria); using our system, biologists can toggle this parameter in the C++ program and emit two alternative protocols to be followed in the laboratory.

For the textual output to appear natural to a human, it is important to keep track of neighboring instructions and to emit code that uses simplifications and pronouns where appropriate. For instance, if two consecutive instructions are measuring fluids into the same container, the second instruction is generated as (for example in Figure 4) "Add 100 *μ*l of Alkaline Lysis Solution". In other words, the container used as the target of the instruction is unambiguous in context, and can be omitted to reduce the verbosity of the text.

In further improving the readability of textual instructions, we had an interesting point of departure from standard programming language design. Typically, programming language designers aim to define a minimal set of orthogonal primitives that, when composed in sequence, can aptly describe the full range of computations. For example, the instruction "multiply-add" is rarely included in a language, since it can be replaced with two simpler instructions. However, when designing a language to facilitate translation to the English language, it is very difficult to assemble sequences of small primitives into natural English text, especially when they can be summarized by a different high-level phrase.

An example of this phenomenon is that of a mixing table, which frequently appears in biology protocols (see Figure 6). While one could encode a table of mixtures as a series of mix instructions, it would be very difficult to recover the simple structure of the table when translating those instructions back to English. Thus, we introduced a mixing_table instruction in the language (also illustrated by example in Figure 6).

This instruction does not complicate the mapping of BioCoder to automatic platforms, as it naturally decomposes into lower-level mix instructions. However, by encapsulating the high-level semantics, we facilitate the translation to English and simultaneously provide an intuitive and concise interface for programmers to use.

Other examples of compound instructions include combine_and_mix, incubate_and_mix, and mount_observe_slide.

#### Timing Constraints

Timing constraints are an important part of biology protocols. Execution of a protocol may require a minimum delay between steps (cell growth, enzyme digest, denaturation, etc.), a maximum latency between steps (avoid precipitation, photobleaching, etc.), or a precise interval between steps (regular measurements, synchronized steps, etc.) We capture these constraints using the wait and time_constraint instructions in BioCoder. Constraints may be expressed relative to another instruction (e.g., wait 1 minute until the next step), or can be attached to a container (e.g., wait 1 minute before any instruction uses this container). Attaching constraints to containers allows precise timing across procedure boundaries, which is otherwise difficult to specify in terms of instruction pairs. We also introduce a use_or_store instruction, which indicates that a container should be stored at a certain temperature (e.g., on ice) if it is not used within a given amount of time. The store_until instruction also allows the timing to depend on a predefined list of events.

#### Implementation of BioCoder

As mentioned previously, BioCoder is implemented as a C++ library that is open source and freely available [19]. The library consists of 5,800 non-comment non-blank lines of C++ code, which is richly documented using the Doxygen [22] tool (there are 2,400 lines of comments and embedded documentation). To simplify the system, most of the source base is valid C code, with all of the BioCoder functions existing in a global namespace. Users can easily extend the system by adding new types or functions to this list.

We depend on the features of C++ in two ways. First, types such as Volume are implemented as a class hierarchy, where the Volume base class is extended by subclasses that represent mimimum, maximum, approximate, symbolic, and ranges of volumes; a similar class hierarchy exists for the Time and Speed types. Object inheritance enables all BioCoder functions to accept different kinds of volumes, times, and speeds, without having to deal with each type separately. Second, we utilize function overloading in C++ to provide many different versions of the same BioCoder function (for example, to configure a thermocycler for various programs). We believe this simplifies usage of the library by non-expert programmers.

### Validation of the Language

However appealing the design of a programming language, the ultimate impact is dictated by its ability to concisely describe the programs of interest, as well as its ease of use by programmers. Evaluating a programming language for biology protocols poses unique challenges, because - unlike computer programs - there does not yet exist a commodity platform that can be used to automatically execute a protocol and check that its output is correct. Thus, we rely on human validation of coded protocols, both via execution in the laboratory as well as manual inspection, to show that BioCoder provides a complete, unambiguous, and correct description.

Our evaluation of BioCoder proceeds in three steps. First, we show that it is possible to describe a broad range of molecular biology protocols in the language. Then, we argue that the formal protocols are correct using evidence from laboratory experiments, manual inspection, and automated tools. Finally, we explore the usability of the language for real biologists, by observing their first experience implementing protocols in BioCoder.

#### Benchmark Suite

**Figure 7:**
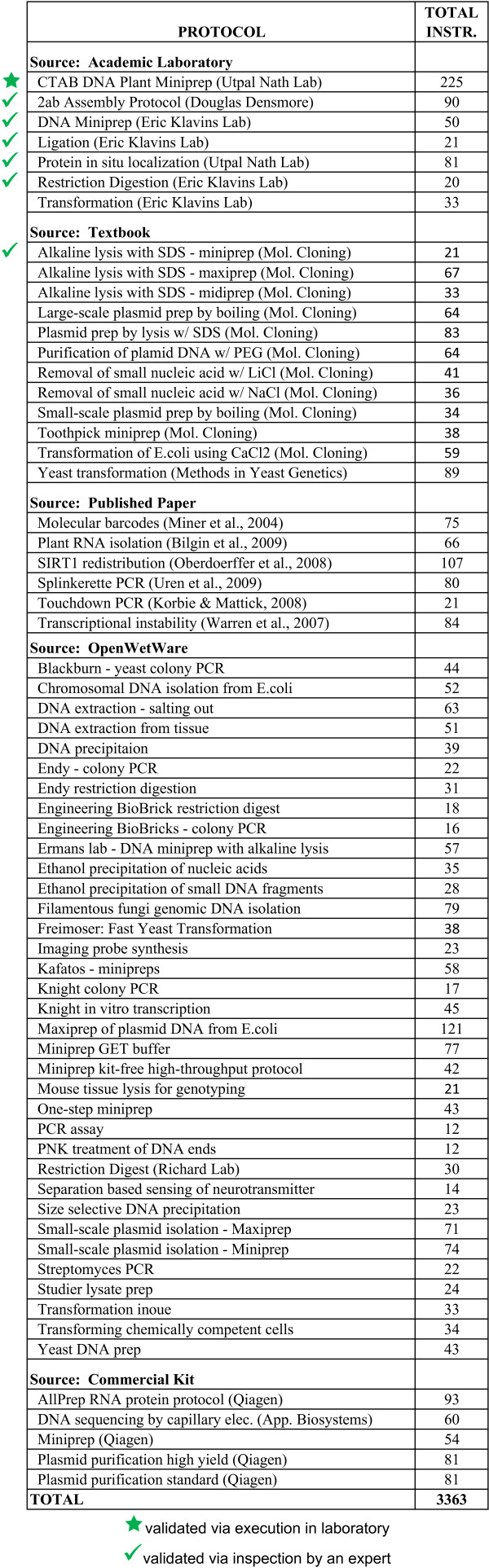
**BioCoder benchmarks**. The number of BioCoder instructions used in each benchmark is shown at right.

The BioCoder benchmark suite appears in Figure 7. We have implemented a total of 66 protocols in the language, drawing from diverse sources such as textbooks, academic laboratories, published papers, commercial kits, and http://OpenWetWare.org (an online wiki for sharing biology protocols). Protocols range in size from 12 BioCoder instructions (PNK Treatment of DNA ends) to 225 BioCoder instructions (CTAB DNA Plant Miniprep), with an average length of 52 instructions. The eleven protocols drawn from the *Molecular Cloning *textbook rely heavily on a shared library of common procedures such as phenol chloroform extraction and ethanol precipitation; these functions consist of 83 BioCoder instructions and are not included in the table. The suite represents over 6600 lines of code.

**Figure 8:**
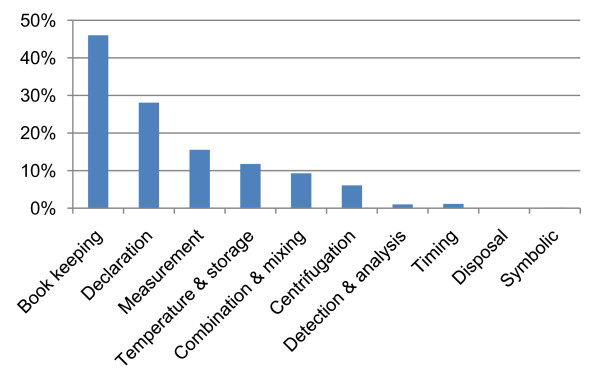
**Instruction usage**. Breakdown of BioCoder instructions as used in our benchmarks.

The usage of BioCoder instructions according to their functionality is shown in Figure 8. Approximately 62% of instructions are used for either book keeping (indicating new steps) or declaring resources. Of steps that translate to experimental actions, the most common are measurement (13%), temperature and storage (10%), combination and mixing (8%), and centrifugation (5%). Our current benchmarks make sparser use of instructions for detection, timing, disposal and symbolic manipulation.

*Comparison to EXACT*. Our "yeast transformation" protocol was selected to enable a comparison to EXACT [9], which uses the same protocol as a case study. Both versions of the protocol are implemented from the original source [23]. While we implement the entire protocol, the EXACT protocol (available online [24]) covers only the first half, which is the preparation of competent cells; thus, we restrict our comparison to that portion.

The most striking difference between BioCoder and EXACT is the verbosity of the description: EXACT requires 704 (non-blank) lines to describe the protocol, while BioCoder requires 46. While both languages provide a complete description of the protocol, EXACT provides more fine-grained steps; for example, it details the preparation of YPD agar and yeast colony plates, whereas BioCoder uses a single "inoculate" instruction. One instruction in BioCoder that is currently missing from the EXACT description is that of alternative steps; in this protocol, BioCoder can express the option of inoculating with either YPD or SC medium.

We view BioCoder as being complementary to EXACT. In fact, the EXACT authors note that "we would like a tool that can translate from a fully specified EXACT protocol into a summarized human-friendly readable format" [9]. BioCoder represents one such design point. In future work, it could be interesting to consider closer integration between the two languages.

#### Validation in the Laboratory

To show that BioCoder provides a faithful representation of a protocol, we conducted an end-to-end experiment whereby a student in a biology laboratory conducted a new, non-trivial protocol while using the BioCoder version as her only reference. Our specific methodology was as follows:

1. We obtained from an academic biology laboratory a customized description of a protocol for manipulating plant DNA. The lab has been using this protocol successfully for several years.

2. We translated the protocol into BioCoder, and automatically generated a human-readable version. We sent this protocol back to the laboratory.

3. An undergraduate intern, who had never conducted protocols on plants, executed the protocol using the BioCoder description as her only reference.

The protocol used for this exercise (labeled "CTAB DNA Plant Miniprep" in Figure 7) is the most complex in our benchmark suite. It involves extracting plant DNA, amplifying a gene, cloning the gene into a plasmid, transformation of bacterial cells with the plasmid and finally, bacterial plasmid miniprep. It requires approximately one week to execute in the laboratory.

The result of our experiment was successful: by following only the BioCoder protocol, the student obtained a valid result, comparable to previous interns who had access to the original description. (We relied on the judgement of the supervising professor to gauge whether the student's progress was comparable to prior interns.) This provides evidence that the BioCoder description of the protocol captured all of the details necessary to execute it in the laboratory.

It is important to emphasize that the student conducting the experiment did not have specific prior knowledge of the protocol under consideration. The student was a second-year undergraduate, and while she had done similar protocols with bacteria, this was her first exposure to manipulating plant DNA. While the student did have access to a mentor in the laboratory, the mentor indicated that the student did not have any confusion with respect to the protocol (her questions focused on how to operate laboratory equipment, understanding the rationale for adding specific reagents, or checking whether certain results were expected).

#### Validation via Manual Inspection

In addition to executing a protocol in the laboratory, for seven protocols we asked a third-party expert (in most cases the original author of the protocol) to inspect the text generated by BioCoder and to compare it to the original. As in the previous section, we obtained the original protocol description from a laboratory and translated it to BioCoder ourselves, before generating a human-readable version for inspection by the expert.

In all cases, the experts deemed the BioCoder version to be a complete and faithful representation of the original, and would not object to using it as the primary reference. In the case of the protein in-situ localization protocol, our collaborating PI commented: "The protocol reads crisper and easier - will be useful for the beginners."

#### Validation via Automated Tools

When protocols are written as computer programs, it also becomes possible to perform simple consistency checks using automated tools. As a first step in this direction, we implemented an overflow/underflow detector, which aims to determine whether a protocol ever attempts to fill a container beyond its capacity, or to remove more volume from a container than is currently present. This check is simple to perform for a given value of the program parameters, by simulating the protocol's execution while keeping track of the volume present in each container. Some instructions (such as centrifugation) may lead to an unknown volume, in which case both extremes can be tracked to see if there is any risk of overflow or underflow in the laboratory.

We ran this analysis over all of the programs in our benchmark suite, as well as programs implemented by biologists as part of our case study (described in the next section). The analysis reported a subtle bug that was common to all of the biologists' codes for miniprep, as well as our own reference versions. The problem stems from usage of the optional_step instruction: when a fluid is transfered to a new container in an optional step, the program variable pointing to the original container must be updated to point to the new one, so that future instructions will use the right container whether or not the optional step was taken. We had originally omitted this update, which caused subsequent instructions to operate on an empty container. The analysis was very useful in exposing this problem.

The analysis also revealed a bug in our language itself, whereby containers could be initialized to hold a fluid of unknown volume. This has since been fixed by requiring a volume for fluids that accompany a container's initialization.

#### Validating Ease of Programming: A Case Study

In order to judge the usability of BioCoder as a tool for biologists, we performed a small user study in which we asked two undergraduate students (who had never used BioCoder) to translate a set of protocols from English-language descriptions into BioCoder code. The students were both final-year undergraduates in a biology program (with a dual-major in engineering); user 1 described his understanding of the C language as "fair" while user 2 described it as "good". We provided a 1.5-hour training session on BioCoder, including example protocols and their text output. We then held a 1-hour practice session with each user individually, where they implemented an example protocol while asking us as many questions as they liked. Finally, we held a test session, where users had access to the documentation, sample programs, and BioCoder-to-text converter, but were discouraged from asking questions. We asked them to implement two miniprep protocols: one that was generated as the text output from BioCoder, and one that was written by a third-party. We expected that the protocol that was previously emitted from BioCoder would be easier to re-implement.

Overall, the outcome of the study was encouraging, with both users producing a result equivalent to our own implementation for at least one protocol. (We later found that our own implementation had a subtle bug that was also mirrored in the users' codes, as detailed in the previous section.) Apart from this issue, as well as a pair of numerical typos by user 1 (20 *μ*L in place of 50 *μ*L, 2 minutes in place of 5 minutes), both users implemented a correct version of the first protocol (miniprep as emitted by BioCoder). The BioCoder instructions chosen by the users were exactly the same as our reference version, with one exception: user 1 called "vortex" in place of "resuspend" (these have equivalent meanings for automation, though produce different text outputs). We also noticed minor differences in parameters, though we have since removed the ambiguity from the language ("ice cold" vs. "on ice", and adjusting options for drying pellets).

The second miniprep protocol was more challenging, because it originated from an original source rather than from BioCoder. For this protocol, user 2's implementation was equivalent to our own, though user 1 substituted a comment instruction for a centrifuge instruction, which would prevent his protocol from running correctly on an automated platform. It seemed that user 1 misunderstood the comment instruction, as he also misused it to declare section titles of the text output ("reagents", etc.) The users' instructions also had other differences from our reference version, including use of store_until in place of dry_pellet and store_for in place of incubate. However, as in the case of the first protocol, these differences would only affect the text output of BioCoder, and still represent an equivalent protocol as far as automation goes.

In terms of time required, user 1 spent approximately 2 hours on each of the protocols, while user 2 (being more proficient in C) required 1 hour and 10 minutes for each. While this is somewhat slow, neither user felt overwhelmed by the language, and they both expected that their proficiency would improve with time.

### Applications of the Language

Independent of the question of validating the language is that of application: if BioCoder does provide a faithful representation of protocols, how could it offer benefits to biologists? We provide early evidence of gains in standardization and automation, and describe future applications in laboratory information systems and education.

#### Standardization

**Figure 9:**
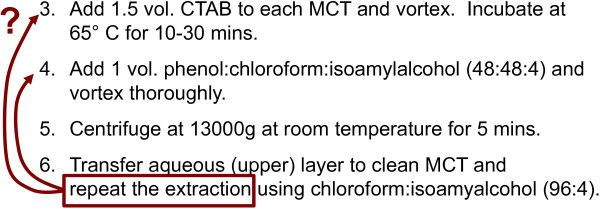
**Resolving ambiguity**. Example ambiguity in a laboratory protocol. While biologists often interpret step 6 as referring to step 4, the intended reference is to step 3.

Thus far, our experience with BioCoder has led us to expose and fix three bugs and ambiguities in real laboratory protocols. The first of these (excerpted in Figure 9) is the protocol for plant DNA extraction, which was being used internally in the laboratory of an academic partner. Step 6 of the protocol is ambiguous in saying to "repeat the extraction", as one could interpret this as a jump to either step 3 or step 4. In an informal poll of dozens of biologists, the common interpretation is to return to step 4; however, upon checking with the original author of the protocol, it turns out that one needs to return to step 3 instead. This is an important ambiguity that could change the outcome of the protocol, yet is entirely due to a simple, unintended slip in English-language wording. Implemented in BioCoder, the task is completely unambiguous, as there is no instruction for "repeating the extraction".

The second ambiguity arises from a protocol for plant DNA extraction [25], where Step 7 instructs one to "wash with chilled 70% ethanol". The undergraduate student who was performing this step (the same one who executed our protocol in the laboratory) interpreted this to mean that the tube containing a pellet should be filled with ethanol and drained immediately; however, the actual method requires resuspension of the pellet via vortexing, followed by centrifugation and draining. A BioCoder description of this step leaves no room for mis-interpretation, as the vortexing and centrifugation are explicit.

Finally, we found two minor bugs in protocols from an academic lab when translating them to BioCoder. In a transformation protocol, a microcentrifuge tube was mis-labeled as a spectrometry cuvette, and in a miniprep protocol, a 1.5 mL tube was mis-labeled as a 1.5- *μ*L tube. These protocols had been used in an undergraduate class, which makes it surprising that the error was not reported (we do not know if it caused confusion in the class).

In order to have a broader impact on protocol standardization, we have taken an active role in http://OpenWetWare.org, a community wiki for sharing biology protocols. In the molecular biology category, we have posted a BioCoder version and corresponding text output for every non-trivial protocol that is not missing key details (33 in total). We hope to engage with the community to make robust and standardized protocols a reality.

#### Automation

One of the most compelling applications of BioCoder is to enable fully-automatic execution of protocols on sophisticated platforms (such a microfluidic chips) in the future. We showed that such automation is tractable in prior work, where we defined a simpler language (BioStream) and executed it automatically on diverse microfluidic chips [14]. BioStream contained only mixing, storage, detection, and I/O primitives, which are fully supported by many microfluidic chips today. We anticipate that as the capabilities of such chips evolve, the task of mapping BioCoder for automatic execution will not be beyond reach.

#### Future Applications

We envision several applications of BioCoder beyond those explored in this paper. One benefit of expressing protocols as a computer program is that they are amenable to automatic scheduling, which could help improve the utilization of shared equipment in a laboratory setting. Our analysis engine can predict the times at which a protocol will demand use of each resource - for example, the thermocyclers, centrifuges, and microscopes - which could aid researchers in reserving time on these machines (or checking for their availability). In a high-throughput setting, automatic scheduling could also be used to optimize the timing of parallel experiments, e.g., to ensure that all equipment in a pharmaceutical corporation is fully utilized.

While we have focused on the usage of BioCoder in the context of research, we also foresee several applications in education. BioCoder can be used to emit different descriptions of the same protocol for different levels of students. For example, for beginners one might generate a detailed text protocol, including all comments and perhaps tutorial information on each step (PCR, centrifugation, etc.). As students progress, the tutorials and hints can be removed, and the graphical protocol may serve as the best summary. BioCoder can also be used to do revision control on protocols; students can calculate an automatic diff between their protocols and their peers', and can track (or rollback) the changes made to their own methods as they evolve over time. It may also be possible to use computers at the lab bench to interactively coach students through their protocols, assist them in understanding or troubleshooting key steps, and enable educators to track their progress.

## Conclusions

We have presented BioCoder, a language and system for describing biology protocols in a form that is suitable for both standardization and automation. To the best of our knowledge, BioCoder is the first programming language that expresses realistic molecular biology protocols in logical terms without undue dependence on the execution platform. We described the key lessons learned in designing the language, including standardization of ad-hoc language, separation of comments from instructions, support for symbolic volumes, ensuring readability of text output, and support for timing constraints. We validated the completeness of the language by implementing over 65 protocols, and demonstrating the first (to our knowledge) end-to-end experiment that was conducted by a human using a programmed protocol as reference. A small user study suggests that BioCoder is accessible to undergraduate students. We look forward to maintaining an open development process and working with the community to find practical applications for BioCoder in improving the reproducibility and automation of the next generation of biology protocols.

## Competing interests

The authors declare that they have no competing interests.

## Authors' contributions

VA and WT jointly designed the BioCoder language. VA implemented and refined the language (as well as the example protocols) and evaluated it via user studies. WT led the preparation of the manuscript. Both authors read and approved the final manuscript.
